# Beep Tones Attenuate Pain following Pavlovian Conditioning of an Endogenous Pain Control Mechanism

**DOI:** 10.1371/journal.pone.0088710

**Published:** 2014-02-13

**Authors:** Raymonde Scheuren, Fernand Anton, Nathalie Erpelding, Gilles Michaux

**Affiliations:** 1 Laboratory of Psychobiology and Neurophysiology, Integrative Research Unit on Social and Individual Development, University of Luxembourg, Luxembourg, Grand-Duchy of Luxembourg; 2 P.A.I.N. Group, Boston Children’s Hospital, Waltham, Massachusetts, United States of America; 3 Department of Anesthesia, Harvard Medical School, Boston, Massachusetts, United States of America; 4 Institute of Health Promotion, St Theresa Clinic, Luxembourg, Grand-Duchy of Luxembourg; University of Sydney, Australia

## Abstract

Heterotopic noxious counter-stimulation (HNCS) is commonly used to study endogenous pain control systems. The resulting pain inhibition is primarily based on spinal cord-brainstem loops. Recently, functional imaging studies have shown that limbic structures like the anterior cingulate cortex and amygdala are also implicated. Since these structures are involved in learning processes, it is possible that the HNCS-induced pain inhibition may depend on specific cues from the environment that have been associated with pain reduction through associative learning. We investigated the influence of Pavlovian conditioning on HNCS-induced pain inhibition in 32 healthy subjects by using a differential conditioning paradigm in which two different acoustic stimuli were either repeatedly paired or unpaired with HNCS. Series of noxious electrical pulse trains delivered to the non-dominant foot served as test stimuli. Diffuse noxious inhibitory control (DNIC)-like effects were induced by concurrent application of tonic HNCS (immersion of the contralateral hand in ice water). Subjective pain intensity and pain unpleasantness ratings and electromyographic recordings of the facial corrugator muscle and the nocifensive RIII flexion reflex were used to measure changes in pain sensitivity. HNCS induced significant pain and reflex inhibitions. In the post-conditioning phase, only the paired auditory cue was able to significantly reduce pain perceptions and corrugator muscle activity. No conditioned effect could be observed in RIII reflex responses. Our results indicate that the functional state of endogenous pain control systems may depend on associative learning processes that, like in the present study, may lead to an attenuation of pain perception. Similar albeit opposite conditioning of pain control mechanisms may significantly be involved in the exacerbation and chronification of pain states.

## Introduction

Endogenous pain control systems include mechanisms like descending inhibition, stress-induced analgesia [1] and diffuse noxious inhibitory controls (DNIC) [2]. In humans, DNIC has also been referred to as counter-irritation analgesia or conditioned pain modulation [3]. It relates to the fact that pain present in one region of the body may be attenuated by an additional pain stimulus applied to another body region. Classically, DNIC appears upon heterotopic noxious counter-stimulation (HNCS) and is increasingly used as a model to study human endogenous pain control mechanisms in both experimental [4], [5], [6] and clinical studies [7], [8].

DNIC-related analgesia was originally studied in animals by focusing mainly on spino-bulbo-spinal pathways [9], [10]. More recently, functional magnetic resonance imaging (fMRI) studies in humans have shown that cerebral structures like the anterior cingulate cortex and the amygdala contribute to HNCS-induced hypoalgesia [11], [12]. Interestingly, these limbic regions have also been found to be involved in learning processes [13], [14]. It is thus conceivable that endogenous pain control systems may be influenced by cues from the environment that have been acquired through associative conditioning. The finding that stress-induced analgesia can be successfully conditioned [15] provides further support for the assumption that associative learning processes may influence pain processing mechanisms and hence possibly play a role in the development of chronic pain (for review see [16]).

Of particular interest within the phenomenon of HNCS-induced hypoalgesia is the enduring effect of therapeutic procedures like acupuncture or transcutaneous electrical nerve stimulation (TENS). DNIC-like processes are thought to mediate at least partially this effect [17], [18]. However, DNIC-related pain inhibition only lasts several minutes [10] whereas the therapeutic efficacy of acupuncture and TENS may persist for hours or even days [19], [20]. Hypothetically, this discrepancy may be attributable to associative learning of initially neutral cues from the environment that may serve as conditioned stimuli for the induction of long-lasting hypoalgesic effects.

The present study was aimed to demonstrate that HNCS-induced pain inhibition can be successfully conditioned. To measure endogenous pain inhibition based on the counter-stimulation and the conditioning procedure, we collected subjective pain intensity and pain unpleasantness ratings and objective physiological parameters of nociception and hyperalgesia like electromyographic (EMG) activity related to facial corrugator muscle- (frowning or brow lowering reflex) and to nocifensive RIII flexion reflex (withdrawal reflex) activity of the biceps femoris muscle, respectively. The corrugator muscle activity is mostly recorded as a measure of primarily negative facial expression while experiencing pain [21]. The RIII reflex is correlated with pain threshold and is commonly used as a tool for the study of pain mechanisms and for the evaluation of treatment [22], [23], [24]. Since we could confirm that psychophysical and psychophysiological pain-related responses were attenuated following the respondent conditioning procedure, the above mentioned main goal of the study was achieved.

## Materials and Methods

### Participants

Participants were recruited among the students and the staff of the University of Luxembourg and received financial compensation. Volunteers with a history of chronic pain, cardiovascular, dermatological, neurological, and psychiatric disorders were excluded from the study. Only those subjects tolerating the cold pressor test for at least 1 min during the assessment of their pain threshold and tolerance level to ice-water immersion prior to the experiment (cf. experimental protocol) were allowed to participate in the study. At the same time point, the participants had to reach pain intensity ratings of at least 2 on a verbally anchored numerical rating scale (NRS; 0–10; 0 = *no pain*, 10 = *worst pain imaginable*; pain ratings were done by increments of 1.0 or 0.5 decimals on the 0–10 NRS) to make sure that the cold pressor test could be used as HNCS. They also had to show an HNCS-induced pain reduction of at least 5% in the pre-conditioning baseline (BL) 2 (i.e. the BL2 stimulation block was characterized by three electrical stimulation series and a simultaneous application of the cold pressor test serving as HNCS) and had to tolerate electrical stimulation during the RIII threshold delineation. Since hypertension has been shown to be associated with lower pain sensitivity [26], only normotensive participants were included (<140 mmHg systolic and 90 mmHg diastolic; manometrically assessed).

Among the 53 recruited participants, 21 subjects could not participate in the experiment, either because the DNIC-effect could not be triggered during BL2 or because they did not tolerate the electrical stimulation intensity during the RIII threshold assessment. A final sample of 32 healthy drug-free subjects (11 female and 21 male; 29 right- and 3 left-handed; age range 18–39 years, median = 23 years) gave informed written consent to participate in the study. As a cover story, participants were informed that they were taking part in an experimental study investigating the relationship between pain and cardiovascular parameters and that the auditory cues merely indicated the duration of the stimulation sequences. Experimental protocols are in line with ethical guidelines of the International Association for the Study of Pain (IASP) [25] and were approved by the National Research Ethics Committee (ref. 1102–59).

### Material and Equipment

Phasic electrical stimuli were provided by a pulse generator (A 310 Accupulser, World Precision Instruments, USA) and were delivered through a constant-voltage-stimulator (Unipolar Pulse STM200, BIOPAC Systems, Inc., USA) [27], [28]. Stimulation was applied through two convex tin electrodes (diameter 0.5 cm; EL350S; BIOPAC Systems Inc., USA) placed 2 cm apart on an acrylic bar. The electrodes were fixed with an adhesive strip posterior to the ankle of the contra-lateral (non-dominant) foot, at the height of the sural nerve. The ankle was flexed at 90° and the knee at 130°. Skin impedance at the foot was measured with a Multimeter Analog HM-120 BZ (Hung Chang Co. Ltd.; Seoul, South Corea) and had to remain below 10 kΩ.

The RIII reflex threshold was assessed with a modified staircase method [23], [29]. Single electrical pulses (1 ms) of increasing strength (ranging from 0.5–3 V) were delivered until the first RIII reflex response emerged. The threshold intensity was considered to be reliable when 2–3 repetitive stimuli yielded stable EMG responses exceeding an integrated area of 100 µV*s [30]. RIII reflex-eliciting stimulation intensity was individually adjusted and fixed at max.110% reflex threshold to preclude pain at tolerance level during the wind-up procedure.

**Figure 1:**
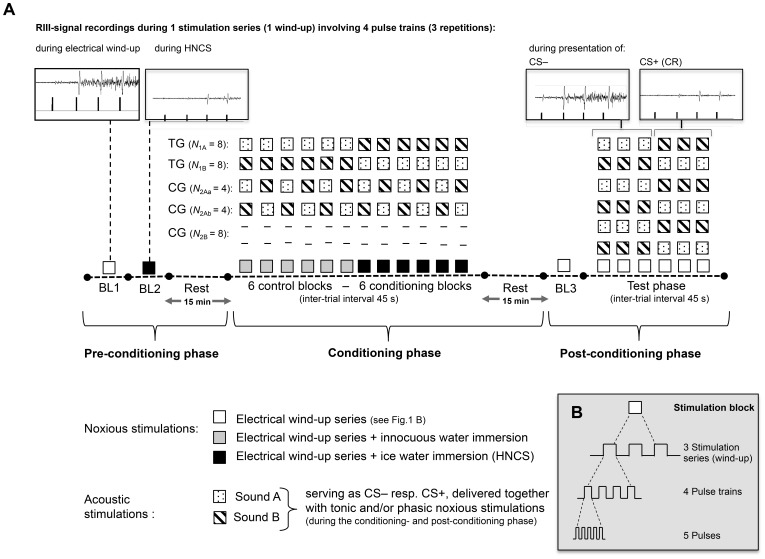
Experimental protocol. Abbreviations: HNCS = heterotopic noxious counter-stimulation, UCR = unconditioned response, CS = conditioned stimulus, CS– = unpaired conditioned stimulus, CS+ = paired conditioned stimulus, CR = conditioned response, TG = test group (*N_1_* = 16), CG = control group (*N_2_* = 16), BL = baseline. (A) Stimulus presentations during the pre-conditioning, conditioning and post-conditioning phases (see further details in the text. (B) Electrical stimulation sequences delivered over each stimulation block.

During the experimental trials, electrical stimulation consisted in rectangular pulse trains (pulse width: 25 ms, repetition rate: 200 Hz, 5 pulses of 1 ms each) [31], [24]. These pulse trains were presented in series of four at an inter-stimulus interval (ISI) of 500 ms to induce temporal summation of the nocifensive RIII reflex [31], [23], [24] and psychophysical pain responses. This paradigm was chosen to have a pain marker that is not influenced by distraction effects [32]. The duration of one wind-up series was 1.6 s. In each stimulation block, three of these series were delivered at intervals of 25 s to avoid habituation of the stimuli. The total duration of the three stimulation series and the respective intervals was ±55 s. A detailed overview of the electrical stimulation paradigm is displayed in Fig. 1B. Specimen of RIII-signal recordings are depicted in Fig. 1A.

HNCS consisted in the immersion of the dominant hand up to the wrist in ice water for 75 s [33]. The water was kept at a constant temperature of approx. 2°C using an external chiller (Aqua Medic GmbH, Germany). For the tepid water control condition, a commercially available submergible heater and an external digital control device (T-controller T2001 HC, Aqua Medic GmbH, Germany) were used to keep water temperature constant (32±2°C).

All electrical and acoustic stimuli were controlled via E-Prime presentation software (Psychology Software Tools Inc., USA).

### Psychophysiological Recording

Physiological activity was continuously recorded with an MP150 Data Acquisition System (BIOPAC Systems Inc., USA).

EMG activity of the facial corrugator supercilious muscle (for measuring frowning responses) and the biceps femoris muscle (for assessing RIII reflex responses) was recorded with an EMG100C amplifier (both with 500 Hz low and 10 Hz high-pass filter and a signal gain of 500). For the RIII reflex measurement, two shielded disposable and pre-gelled Ag-AgCl electrodes (diameter 24 mm, H124SG, Kendall Electrodes) were placed at the non-dominant upper leg, over the short head of the biceps femoris muscle (distance between electrodes 20 mm) [30], [34]. Recordings were only initiated when the impedance was below 5 kΩ. The same type of electrodes was also used for corrugator muscle activity recording. The electrodes were fixed 15 mm apart over the left eyebrow in parallel to the muscle midline [35]. Before application of the EMG recording and stimulation electrodes, the skin at the leg, foot, and forehead was cleaned with ethanol and abraded. The electrode placement area on the leg was shaved when necessary.

Beat-to-beat BP was measured by analyzing the timing and amplitude of the primary left ventricular ejection pulse as well as the arterial pulse reflections at the wrist of the non-dominant arm (NIB P100A; Medwave Vasotrac APM205A). A standard precordial lead II electrocardiogram (ECG) (ECG100C; 0.5 Hz high pass filtering, R-wave output mode, signal gain 500) was performed using disposable pre-gelled Ag-AgCl electrodes (diameter 35 mm, EL502, Biopac Systems) placed below the right clavicle and below the left lower rib. Pulse and ECG recordings were used to compute continuous HR.

Electrodermal activity was assessed with two domed Ag-AgCl electrodes (diameter 6 mm, SS3LA, Biopac Systems) filled with isotonic paste (containing 0.5% saline in a neutral base). The electrodes were attached to the mid-phalanx of the third and the fourth finger of the non-dominant hand. The signal was processed through a constant voltage (0.5 V) coupler (GSR100C, 1.0 Hz low pass filtering, signal gain 5 µS/V).

Subjects were grounded through an unshielded disposable Ag-AgCl electrode (diameter 24 mm, H124SG, Kendall Electrodes) positioned at the midpoint of the left calf (non-dominant leg.

### Experimental Protocol

The protocol corresponded to a randomized controlled trial. Experimental sessions were based on a differential conditioning paradigm and comprised a pre-conditioning (i.e. baseline), a conditioning (i.e. acquisition) and a post-conditioning (i.e. test) phase. The experimental stimulation blocks were identical for all experimental groups, except for the differential procedure during the conditioning phase. The experimental procedure is summarized in Fig. 1A. Each subject participated in a single session lasting about two hours. Experiments took place in a temperature-controlled room (approximately 22°C) and were all performed by the same investigator.

Prior to the beginning of the experiment, the participants’ pain threshold and tolerance level to ice-water immersion was measured. The subjects immersed their dominant hand into the ice water bath over a period of 1 minute and rated the induced pain intensity on a 10-point NRS in 10 s intervals. Subsequently, all electrodes and sensors were attached (see material and equipment section). Participants were then given a 5 min rest before the RIII reflex threshold was determined. For this purpose, single electrical pulses of increasing intensity were applied until the electrical stimulation reliably induced an RIII reflex. Pain intensity ratings of the applied pulses were assessed simultaneously.

Whereas the objective psychophysiological responses to the phasic electrical test stimuli were continuously measured online during the pre- and post-conditioning phases, the subjective pain intensity and pain unpleasantness perceptions were assessed only at the end of each electrical stimulation series (wind-up; 3 × per stimulation block), but throughout the whole experiment.

In the pre-conditioning phase, all participants were submitted to two baseline measurements (BL1 and BL2, see Fig. 1A). BL1 involved three electrical stimulation series and BL2 was characterized by a simultaneous application of the cold pressor test serving as HNCS.

For the conditioning and post-conditioning phases, subjects were randomly assigned to the test group *(N_1_* = 16) or to the control group (*N_2_* = 16). Subjects in the test group were exposed to a differential conditioning procedure. Here, two sounds of different frequency were used as conditional stimuli (CS). A common dial phone signal, consisting of a 344-Hz continuous wave, was considered as sound A, whereas a busy phone signal, made up of a 600-Hz interrupted wave, was applied as sound B. Each sound was presented with 65 dB via headphones. To test for response generalization, CS+ salience and habituation, these initially neutral acoustic stimuli were presented in counterbalanced order with regard to their use as CS. Half of the participants in the test group (*N_1A_* = 8) did consequently receive sound A as CS– and sound B as CS+, whereas the attribution of the tones was reversed in the other half of the test group (*N_1B_* = 8), sound B serving as CS– and sound A as CS+. Participants in the test group were randomly assigned to one of these two conditions. During the conditioning phase, the acoustic stimuli were either paired (CS+) or unpaired (CS–) with repeated immersion of the dominant hand into the ice or tepid water bath. In the test group, innocuous tepid water immersion was consistently unpaired with CS– and noxious ice water immersion was always paired with CS+ (see experimental protocol in Fig. 1A).

The conditioning phase started 15 min after the baseline assessments BL1 and BL2 to allow HNCS-induced inhibitory effects to fade out [24]. Subjects in all experimental groups had six neutral (tepid water immersion) and six HNCS (cold water immersion) blocks. Tepid water was used as control condition and was always applied before cold water in order to avoid a potential activation of counter-irritation mechanisms [24]. Tonic noxious stimulation (ice water, HNCS) not only served as trigger to induce DNIC-like effects, but also as unconditioned stimulus (US). Phasic noxious electrical pulses that were applied to the contralateral foot were used as test stimuli. According to the respondent conditioning model, pain sensation- and reflex response alterations upon HNCS constituted the unconditioned response (UR). The inhibition of nociceptive processing induced by CS+ during the post-conditioning phase was considered as the conditioned response (CR).

A bubble sound (50 dB) signaled when to immerse the dominant hand into the water bath. The ice or tepid water exposure as well as the auditory stimulations (CS) always persisted for 75 s. These thermal and acoustic stimuli were initiated and terminated simultaneously. Since pain sensations during the cold-water immersions do not occur immediately but typically show a delay [36], electrical stimulation series (3 wind-up) were applied 20 s after the start of the tonic pain stimulation and lasted in total 55 s. Participants were instructed to lift their hand out of the water bath during inter-trial intervals (period of 45 s). Together with the thermal/acoustic stimulation duration (75 s) and the related ISI (45 s), 120 s (2 min) were required for one stimulation block.

Contrary to the test group, participants in the control group (*N_2_* = 16) were not subjected to any associative learning procedure, but only to unpaired pain stimulations. In order to account for potential confounding (e.g. distraction and alertness due to the presentation of the auditory cues) and sequence effects (e.g. sensitization and habituation due to the repeated stimulus presentations) over the time course of the experiment, the control group was subdivided. Half of the respective participants (*N_2A_* = 8) received the same auditory cues (sound A and sound B) as the test group. These acoustic stimuli were however randomly presented with the tepid or cold-water immersions (i.e. truly random control). To ensure counterbalancing of the sounds, the order A B was presented to half of these *N_2A_* participants (*N_2Aa_* = 4), whereas the other half (*N_2Ab_* = 4) perceived the order B A. The first six acoustic stimuli were unpaired with tepid water, the second six ones were paired with ice water immersions. The second half of the control group (*N_2B_* = 8) was exposed to the same sequence of tonic stimuli as all the other participants, without however receiving any acoustic cues (see Fig. 1A).

The post-conditioning phase started 15 min after the end of the conditioning phase in order to avoid HNCS-related inhibitory hangover [24]. The purpose of the post-conditioning phase was to investigate associative learning effects that were acquired during the conditioning phase. Before the actual start of the post-conditioning phase, a final BL measurement (BL3) was performed to assess pain intensity, pain unpleasantness and physiological parameters while administering only electrical stimulation. In six post-conditioning test trials, phasic electrical stimuli were simultaneously presented with auditory cues. The counterbalanced order of the auditory cues was the same for the test group and the control group. To avoid possible contamination effects from the CS+ onto the CS– [24], CS– was presented in the first three trials and CS+ in the last three trials (cf. Fig. 1A). The experimental session ended with the removal of all electrodes and verbal debriefing of the participants.

### Data Analyses

Pain intensity, pain unpleasantness, corrugator and RIII reflex activity were analyzed in response to electrical stimuli. Psychophysical responses were evaluated for the pre-conditioning-, conditioning- and post-conditioning phases. Corrugator and flexion reflex recordings were only examined in association with pre- and post-conditioning trials. To take into account a potential involvement of baroreceptor reflex mechanisms in the regulation of pain sensitivity [37], [38], BP and HR data were evaluated in periods including (BL2) and in those not including cold-water immersion (BL1, BL3, CS−/CS+ trials). Possible changes in electrical stimulation conditions throughout the experiment were monitored by contrasting electrodermal activity (EDA; in µS) measured during pre-conditioning BL1 and post-conditioning BL3.

AcqKnowledge Software package (BIOPAC Systems Inc., USA) was used for physiological data collection and offline analysis. To assess the corrugator- and RIII reflex activity, integrated EMG was derived from the respective raw data. For analyses of corrugator muscle activity, the EMG data recorded during each ISI (500 ms between two pulse trains) [39] were used and averaged over each stimulation block. The investigation of overall magnitudes of the RIII reflex responses, as well as RIII wind-up ratios was based on the EMG recording periods ranging from 90 to 180 ms following each pulse train ([40], [41], [29]; see specimen RIII waveforms in Fig. 1A). To define the overall RIII magnitudes, all EMG-values recorded during each stimulation block were averaged. Wind-up-induced RIII responses were analyzed for each stimulation series by subtracting the reflex amplitudes obtained in response to the first pulse train from those obtained to the last (4^th^) one. The respective data were then averaged over the 3 stimulation series of each stimulation block and expressed as percent difference (Δ%). Mean (systolic) blood pressure (BP), heart rate (HR) and electrodermal values recorded during the ±1-min stimulation blocks were analyzed.

HNCS-induced changes in pain ratings and pain-related reflexes were computed by plotting differences between the pre-conditioning BL1 (i.e. phasic pain stimulation only) and BL2 (i.e. phasic pain stimulation+HNCS). The CS– and CS+ values of the test phase trials were averaged to identify differences between the post-conditioning BL3 (i.e. phasic pain stimulation only) measures and the post-conditioning CS– and CS+ (i.e. electrical and acoustic stimulation) values, respectively. HNCS and CS-induced changes in pain and reflex responses were depicted as difference (Δ %).

SPSS software (IBM Corp., USA) was used for the statistical analyses of psychophysical and psychophysiological data. Since some corrugator and RIII values exceeded physiologically reasonable measures (probably related to artefacts like movement, electrode contact), we decided to consider them as outliers and excluded them from the statistical analysis. The corrugator data of one participant of the test group and of three participants of the control group were left out. Also the RIII values of one participant of the control group could not be included in the statistical analyses. Technical problems with the blood pressure measurement unit resulted in the loss of cardiovascular data from nine participants. Arithmetic mean and standard error values were used as measures for central tendency and dispersion. The normal distribution of the different variables was verified with the Kolmogorov-Smirnov test. Analyses of variance (ANOVA) for repeated measures and post hoc comparisons (parametric *t*-tests for paired samples) were performed to identify significant differences in pain and reflexes between experimental phases. Greenhouse-Geisser corrections were used in case of violation of the sphericity assumption. In addition, we were interested in possible interactions between the group factor (test and control group) and the repeat factor CS– and CS+ in the post-conditioning phase. We computed 2×(2) ANOVA for all dependent variables to uncover potential significant differences between CS– and CS+ values that were characteristic for the test group, but not for the control group. The potential contribution of blood pressure [26] to differences between the test and the control group and gender-related differential expression of RIII reflex responses [42], [43] were analyzed by computing between-subject ANOVA and post hoc comparisons (independent *t*-tests). Statistical significance was set at p≤0.05 (one-tailed).

## Results

### Psychophysical Data

**Figure 2:**
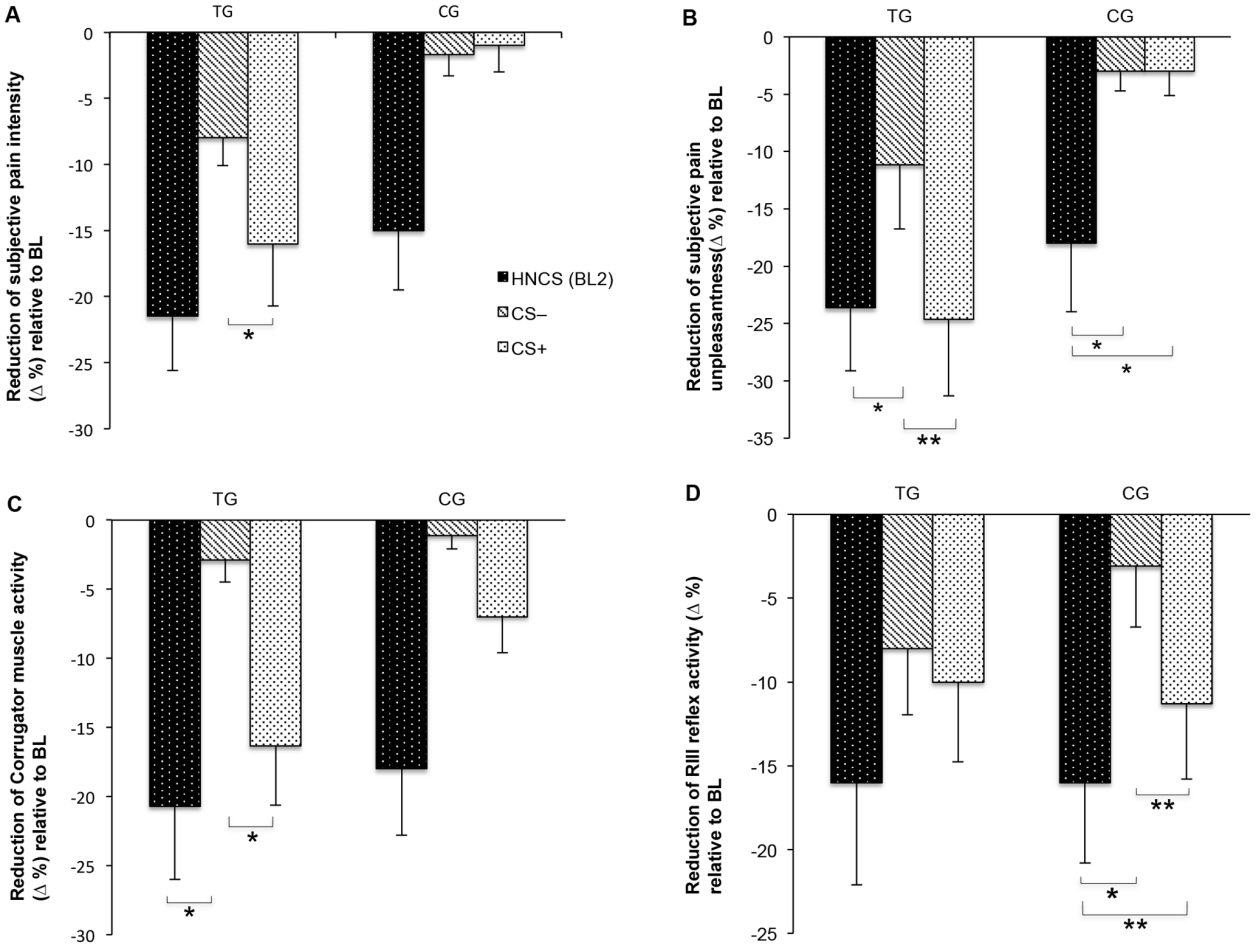
Psychophysical and psychophysiological data of the test group (*N_1_* = 16) and the control group (*N_2_* = 16) during pre-conditioning HNCS (BL2) and post-conditioning CS−/CS+ trials (3 CS– trials; 3 CS+ trials). Pre-conditioning BL2 values were contrasted to pre-conditioning BL1 values (1 trial for each BL). Post-conditioning CS−/CS+ values were contrasted to post-conditioning BL3 values. (A) Pain intensity decrease relative to BL. (B) Reduction in pain unpleasantness relative to BL. (C) Inhibition of corrugator muscle activity relative to BL. (D) Overall magnitude RIII reflex inhibition relative to BL. Abbreviations: TG = test group, CG = control group, HNCS = heterotopic noxious counter-stimulation, BL = baseline, CS– = unpaired conditioned stimulus, CS+ = paired conditioned stimulus, Δ % = percent difference. Results were based on absolute values and were presented as percent difference measures. Arithmetic mean and standard error of the mean (*AM* ± *SEM*) were used as measures for central tendency and variability. For the differences between test phase effects, p-values of *p<0.05 and **p<0.005 were considered as significant and highly significant.

Pain intensity and pain unpleasantness values are represented in Table 1, Table 2, Fig. 2A and Fig. 2B. The Kolmogorov-Smirnov test confirmed a normal distribution of the considered psychophysical variables (all *p*>0.10).

**Table 1:** Psychophysical and psychophysiological data (absolute values).

Outcome measures:	Pain intensityratings NRS (0–10)		Pain unpleasantnessratings NRS (0–10)		Corrugator muscle activity(Frowning reflex)Integrated EMG (µV)		Biceps femoris muscleactivity (RIII flexionreflex) Integrated EMG(µV) Overall RIII magnitude	
Groups:	Testgroup	Controlgroup	Testgroup	Controlgroup	Testgroup	Controlgroup	Testgroup	Controlgroup
	(N_1_ = 16)	(N_2_ = 16)	(N_1_ = 16)	(N_2_ = 16)	(N_1_ = 15)	(N_2_ = 13)	(N_1_ = 16)	(N_2_ = 15)
	*AM ± SEM*	*AM ± SEM*	*AM ± SEM*	*AM ± SEM*	*AM ± SEM*	*AM ± SEM*	*AM ± SEM*	*AM ± SEM*
**Test stimuli^a^ responses:**								
**Pre-conditioning**								
phase:								
BL1	5.4±0.4	5.5±0.4	5.4±0.4	6.3±0.4	0.761±0.10	0.890±0.13	0.138±0.02	0.159±0.02
BL2	4.3±0.5	4.7±0.4	4.3±0.5	5.3±0.5	0.540±0.06	0.622±0.05	0.116±0.02	0.132±0.02
**Conditioning phase:**								
a) Control blocks:								
SB 1	4.4±0.5	5.7±0.5	4.5±0.5	6±0.4				
SB 2	4.7±0.5	5.5±0.5	4.8±0.5	6.2±0.4				
SB 3	4.5±0.5	5.7±0.4	4.7±0.5	6.2±0.4				
SB 4	4.4±0.5	5.1±0.4	4.5±0.5	6.2±0.5				
SB 5	4.4±0.5	5.1±0.4	4.3±0.5	6.1±0.5				
SB 6	4.2±0.5	5.3±0.4	4.2±0.5	6.4±0.4				
b) Acquisition blocks:								
SB 7	2.9±0.5	4.3±0.4	2.9±0.5	5.4±0.5				
SB 8	2.8±0.5	4.4±0.4	3±0.5	5.5±0.5				
SB 9	3±0.6	4.5±0.4	3.1±0.5	5.3±0.6				
SB 10	3.1±0.5	4.5±0.4	3.1±0.5	5.4±0.6				
SB 11	2.9±0.5	4.4±0.4	3.1±0.6	5.3±0.6				
SB 12	2.7±0.5	4.6±0.4	2.8±0.6	5.4±0.6				
**Post-conditioning phase:**								
BL 3	4.8±0.4	5.5±0.4	5±0.5	6.4±0.4	0.688±0.10	0.644±0.10	0.125±0.02	0.101±0.01
Test phase:								
CS–1	4.5±0.5	5.4±0.3	4.5±0.6	6±0.4	0.689±0.10	0.664±0.10	0.123±0.02	0.097±0.01
CS–2	4.4±0.5	5.3±0.3	4.4±0.5	6±0.5	0.683±0.10	0.618±0.10	0.113±0.02	0.097±0.01
CS–3	4.5±0.5	5.4±0.4	4.6±0.5	6.2±0.5	0.671±0.10	0.629±0.10	0.116±0.02	0.097±0.01
Mean CS– (1–3)	4.4±0.5	5.4±0.3	4.5±0.5	6.1±0.5	0.681±0.10	0.637±0.10	0.117±0.02	0.097±0.01
CS+4	4.2±0.5	5.5±0.4	4±0.6	6.2±0.4	0.652±0.10	0.627±0.10	0.117±0.02	0.090±0.01
CS+5	4.1±0.5	5.4±0.4	4.1±0.6	6.2±0.4	0.501±0.07	0.611±0.10	0.111±0.02	0.091±0.01
CS+6	3.9±0.5	5.4±0.4	3.8±0.6	6.1±0.4	0.490±0.07	0.558±0.10	0.107±0.02	0.084±0.01
Mean CS+ (4–6)	4±0.5	5.4±0.4	4±0.6	6.2±0.4	0.548±0.10	0.599±0.10	0.112±0.02	0.086±0.01

a Phasic electrical stimulation. Abbreviations: NRS = Numerical rating scale, EMG = electromyography, AM = arithmetic mean, SEM = standard error of the mean, BL = Baseline, SB = stimulation block, CS– = unpaired conditioned stimulus, CS+ = paired conditioned stimulus. One-tailed *p*-values of *p<0.05 and **p<0.005 were considered as significant and highly significant.

**Table 2:** Psychophysical and psychophysiological statistical overall magnitude data analyses.

Baseline – CS comparisons:								
Pre-conditioning phase: BL1, BL2;								
Post-conditioning phase: BL3, Test phase: Mean CS–, Mean CS+								
Pain intensity ratings	Within-Subjects Effects:			Post hoc comparisons:				
NRS (0–10)	Main effect ‘Pain intensity’							
	(Greenhouse-Geisser correction)			Stimulation block:	Test group (*N_1_* = 16)		Control group (*N_2_* = 16)	
	*F (df)*	*p*	*d*		*t (df)*	*p*	*t (df)*	*p*
		(one-tailed)				(one-tailed)		(one-tailed)
	4.35 (1.99)	.01*	.13	BL1– BL2	5.78 (15)	<.005**	2.78 (15)	.007*
				BL3– mean CS–	1.23 (15)	.12	.97 (15)	.17
				BL3– mean CS+	2.67 (15)	.009*	.12 (15)	.45
				Mean CS– – mean CS+	1.94 (15)	.03*	–.78 (15)	.23
**Pain unpleasantness ratings**	**Within-Subjects Effects:**			**Post hoc comparisons:**				
**NRS (0–10)**	**Main effect ‘Pain unpleasantness’**							
	**(Greenhouse-Geisser correction)**			**Stimulation block:**	**Test group (** ***N_1_*** ** = 16)**		**Control group (** ***N_2_*** ** = 16)**	
	***F (df)***	***p***	***d***		***t (df)***	***p***	***t (df)***	***p***
		**(one-tailed)**				**(one-tailed)**		**(one-tailed)**
	6.87 (2.23)	<.005**	.18	BL1– BL2	4.59 (15)	<.005**	2.53 (15)	.01*
				BL3– mean CS–	–1.54 (15)	.07	.74 (15)	.23
				BL3– mean CS+	3.39 (15)	<.005**	–.06 (15)	.47
				Mean CS– – mean CS+	6.55 (15)	<.005**	–.65 (15)	.26
**Corrugator muscle activity**	**Within-Subjects Effects:**			**Post hoc comparisons:**				
**(Frowning reflex)**	**Main effect ‘Corrugator’**							
**Integrated EMG (µV)**	**(Greenhouse-Geisser correction)**			**Stimulation block:**	**Test group (** ***N_1_*** ** = 15)**		**Control group (** ***N_2_*** ** = 13)**	
	***F (df)***	***p***	***d***		***t (df)***	***p***	***t (df)***	***p***
		**(one-tailed)**				**(one-tailed)**		**(one-tailed)**
	3.33 (2.13)	.02*	.11	BL1– BL2	3.05 (14)	<.005**	2.08 (12)	.03*
				BL3– mean CS–	.28 (14)	.39	.27 (12)	.39
				BL3– mean CS+	1.68 (14)	.05*	.89 (12)	.19
				Mean CS– – mean CS+	1.71 (14)	.05*	1.17 (12)	.13
**Biceps femoris muscle**	**Within-Subjects Effects:**			**Post hoc comparisons:**				
**activity (RIII flexion reflex)**	**Main effect ‘RIII’**							
**Integrated EMG (µV)**	**(Greenhouse-Geisser correction)**			**Stimulation block:**	**Test group (** ***N_1_*** ** = 16)**		**Control group (** ***N_2_*** ** = 15)**	
	***F (df)***	***p***	***d***		***t (df)***	***p***	***t (df)***	***p***
		**(one-tailed)**				**(one-tailed)**		**(one-tailed)**
	9.80 (1.55)	<.005**	.25	BL1– BL2	2.35 (15)	.02*	3.23 (14)	<.005**
				BL3– mean CS–	.28 (14)	.10	1.44 (14)	.08
				BL3– mean CS+	1.68 (14)	.05*	2.95 (14)	≤.005**
				Mean CS– – mean CS+	1.71 (14)	.10	2.63 (14)	.01*

Abbreviations: CS = conditioned stimulus, BL = Baseline, CS– = unpaired conditioned stimulus, CS+ = paired conditioned stimulus, NRS = Numerical rating scale, EMG = electromyography, SB = stimulation block. One-tailed *p*-values of *p<0.05 and **p<0.005 were considered as significant and highly significant.

#### Pain intensity

The initial cold pressor test performed prior to the experimental protocol induced pain intensities gradually increasing over the ±1 min stimulation period. Differences between the first and the last (6^th^) pain intensity ratings on the 10-point NRS ranged in average from 2.6 to 8.1 in the test group and from 2.5 to 7.3 in the control group. Generally, electrical stimulation intensities evoking pain intensity ratings of 3 to 5 corresponded to the RIII reflex threshold.

During BL2 of the pre-conditioning phase, counter-stimulation caused a significant decrease in electrically induced pain intensity in the test group (21% ±4.1; *t_15_* = 5.78, *p*<0.005) and in the control group (15% ±4.5; *t_15_* = 2.31, *p*<0.05) (see Fig. 2A and Table 2). In the post-conditioning (test) phase of the test group, the initially neutral sound that served as paired conditioned stimulus (CS+) was able to inhibit pain (17% ±4.8). This inhibitory effect was comparable to the one that was found for the HNCS itself (see Fig. 2A). The presentation of the unpaired CS– resulted in a lower pain reduction (8% ±2.1). Consequently, pain intensity was rated significantly lower during CS+ than during CS– (*t_15_* = 1.94, *p*<0.05; see Table 2). In the control group, the presentation of the auditory stimuli did not bring essential alterations in pain intensity (CS+: 1% ±2; CS–: 1.5% ±1.6; see Fig. 2A). We did not observe any significant difference between CS– and CS+ presentation (*t_15_* = –0.78, *p*>0.05) (see Table 2).

#### Pain unpleasantness

Pain unpleasantness was significantly inhibited by HNCS in all experimental groups (24% ±5.4 for the test group; 18% ±5.9 for the control group; see Fig. 2B). In addition, we observed a pronounced conditioning effect in the test group exhibiting a pain unpleasantness reduction of 25% (25% ±6.6) that was typical for CS+, whereas CS– produced significantly less attenuated pain unpleasantness sensations (11% ±5.6; see Fig. 2B; *t_15_* = 6.55, *p*<0.005; see Table 2). In contrast to this finding, pain unpleasantness ratings in the post-conditioning phase remained almost unaltered under control condition (CS+: 3% ±2.1; CS–: 3% ±1.7; see Fig. 2B). The difference between pain ratings related to CS+ and CS– was not significant (*t_15_* = –.65, *p*>0.05; see Table 2) in the control group.

The 2x(2) ANOVA analyses of pain rating data did not reveal a significant interaction between the group and the repeat factor CS– and CS+. In these tests, a substantial difference in pain intensity [*F*(1,60) = 2.33, *p*>0.05] and pain unpleasantness [*F*(1,60) = .35, *p*>0.05] responses during CS– and CS+ could not be revealed for the test group and for the control group. A significant main effect of group on the sensory-discriminative [*F*(1,60) = 6.77, *p*<0.05] and the affective-motivational [*F*(1,60) = 12.7, *p*<0.005] component of pain sensations was observed.

### Psychophysiological Data

All psychophysiological data are summarized in Tables 1, 2, 3 and 4. Δ % values are shown in Fig. 2C and Fig. 2D. The two examined objective measures are normally distributed (all *p*>0.10).

**Table 3:** Systolic blood pressure measures (mmgH).

Experimental phases	Test group	Control group	Between-Groups Effects:		Post hoc comparisons	
(Stimulation blocks):	(N_1_ = 11)	(N_2_ = 12)			Test group– Control group:	
	AM ± SEM	AM ± SEM	*F (df)*	*p*	*t (df)*	*p*
				(one-tailed)		(one-tailed)
**Pre-conditioning phase:**						
BL1	13.3±.55	13.3±.64	.05 (1)	.40	–.23 (21)	.40
BL2	15.4±.91	15.4±.66	.00 (1)	.49	–.01 (21)	.49
**Post-conditioning phase:**						
BL3	13.4±.41	12.6±.52	1.42 (1)	.12	1.19 (21)	.12
Test phase:						
CS–1	13.3±.33	12.8±.55	.57 (1)	.22	.75 (21)	.22
CS–2	13.0±.44	12.7±.58	.16 (1)	.34	.40 (20)	.34
CS–3	13.2±.47	12.4±.53	1.29 (1)	.13	1.13 (21)	.13
CS+1	13.2±.39	12.6±.54	1.00 (1)	.16	1.00 (21)	.16
CS+2	13.4±.37	12.7±.54	.00 (1)	.47	–.06 (21)	.47
CS+3	12.9±.50	13.0±.54	.03 (1)	.42	.21 (21)	.42
Mean CS– (1–3)	13.0±.42	12.8±.55	.71 (1)	.20	.84 (21)	.20
Mean CS+ (4–6)	13.1±.39	12.8±.52	.14 (1)	.35	.37 (21)	.35

Abbreviations: AM = arithmetic mean, SEM = standard error of the mean, *df* = degrees of freedom, BL = baseline, CS– = unpaired conditioned stimulus, CS+ = paired conditioned stimulus. One-tailed *p*-values of *p<0.05 and **p<0.005 were considered as significant and highly significant.

**Table 4:** RIII reflex-related wind-up values.

Biceps femoris muscle activity (RIII flexion reflex)						
RIII wind-up measures						
Integrated EMG (µV)						
Test stimuli^a^ responses:						
Experimental phases	Test group			Control group		
(Stimulation blocks):	(N_1_ = 16)			(N_1_ = 15)		
	AM ± SEM			AM ± SEM		
	1^st^ pulse	4^th^ pulse	Wind-up effect	1^st^ pulse	4^th^ pulse	Wind-up effect
			(Δ%)			(Δ%)
**Pre-conditioning phase:**						
BL 1	0.028±0.006	0.041±0.006	43 (Δ%)	0.029±0.005	0.050±0.009	72 (Δ%)
BL 2	0.024±0.003	0.036±0.007	50 (Δ%)	0.026±0.003	0.039±0.006	53 (Δ%)
**Post-conditioning phase:**						
BL 3	0.024±0.004	0.035±0.006	46 (Δ%)	0.020±0.002	0.025±0.003	
Test phase:						28 (Δ%)
CS–1	0.028±0.006	0.037±0.007	34 (Δ%)	0.020±0.002	0.025±0.002	
CS–2	0.027±0.006	0.035±0.006	29 (Δ%)	0.017±0.001	0.028±0.003	26 (Δ%)
CS–3	0.030±0.007	0.035±0.006	17 (Δ%)	0.020±0.003	0.025±0.003	60 (Δ%)
CS+1	0.029±0.007	0.038±0.007	27 (Δ%)	0.018±0.002	0.024±0.003	23 (Δ%)
CS+2	0.028±0.007	0.036±0.006	30 (Δ%)	0.018±0.002	0.026±0.003	35 (Δ%)
CS+3	0.027±0.006	0.031±0.005	25 (Δ%)	0.018±0.002	0.023±0.003	35 (Δ%)
Mean CS– (1–3)	0.028±0.006	0.036±0.006	16 (Δ%)	0.019±0.002	0.026±0.003	40 (Δ%)
Mean CS+ (4–6)	0.028±0.007	0.035±0.006	24 (Δ%)	0.018±0.002	0.0204±0.003	24 (Δ%)

One stimulation block included three phasic electrical stimulation series (wind-up). Each stimulation series comprised four electrical pulse trains (see Fig. 1B). The wind-up effects were calculated by substracting reflex-induced EMG-values obtained in response to the first pulse train from those obtained to the last (4^th^) one. The respective data were then averaged over the three stimulation series of each stimulation block and presented in percent difference (Δ%).

a Phasic electrical stimulation. Abbreviations: EMG = electromyography, AM = arithmetic mean, SEM = standard error of the mean, BL = Baseline, CS– = unpaired conditioned stimulus, CS+ = paired conditioned stimulus.

#### Corrugator muscle activity

Counter-stimulation considerably inhibited corrugator muscle activity in all experimental groups (21% ±5.3 for the test group, 18% ±4.8 for the control group; see Fig. 2C). In the post-conditioning period, CS+ induced a robust reduction of EMG-activity in the test group (16% ±4.3; see Fig. 2C). This decline was significantly more pronounced than the one observed under CS– conditions (3% ±1.6; *t_14_* = 1.71, *p*≤0.05; see Fig. 2C and Table 2). We did not detect any significant CS+ or CS– effect in the control group. The frowning response decreased by 7% ±2.6 in the CS+ condition and by 1% ±1.1 in the CS– condition (see Fig. 2C). There were no significant differences in facial expression between these two conditions in this group (*t_12_* = 1.17, *p*>0.05; see Table 2).

#### RIII flexion reflex

Stimulation intensities ranging from 0.1–9.9 mA (3.3±3.01) were required to evoke reliable RIII reflexes.

In the test group, the overall RIII reflex magnitude was reduced by 16% ±6.1 when electrical stimuli and HNCS were applied concurrently (see Fig. 2D). Under the same conditions, the control group also displayed a reduction in RIII activity of 16% ±4.8; see Fig. 2D). In the post-conditioning phase, the presentation of CS+ induced reductions of reflex activity of 10% ±4.8 in the test group and of 11% ±4.5 in the control group. Reflex attenuations of 8% ±3.9 in response to the CS– were observed in the test group and of 3% ±3.6 in the control group (see Fig. 2D). CS– and CS+ induced reflex responses were not significantly different (*t_15_* = 1.3, *p*>0.05; see Table 2) in the test group. In the control group, CS+ was however accompanied by a significantly more pronounced attenuation of the reflex as compared to CS– (*t_14_* = 2.6, *p*<0.05; see Table 2).

In the test group the analysis of the RIII-related wind-up effects throughout all baseline- and test phase trials revealed average increases ranging from 24% to 50% when comparing the 1^st^ and the 4^th^ pulse train of a stimulation series. In the control group, wind-up related increases in reflex activity were realized in all the stimulation blocks of interest and ranged from 28% to 76%. Absolute values related to the 1^st^ and the 4^th^ pulse train, as well as Δ % measures are shown in Table 4.

No sex-related differences in RIII reduction were observed in our sample (all *p*>0.10).

When comparing the test and the control group with respect to the CS– and CS+ related corrugator and RIII responses of the post-conditioning phase, the 2Δ (2) analyses of variance did not disclose any significant interaction for corrugator [*F*(1,52) = .33, *p*>0.05] and RIII measures [*F*(1,58) = .03, *p*>0.05]. A group main effect on the frowning [*F*(1,52) = .002, *p*>0.05] and withdrawal [*F*(1,58) = 2.15, *p*>0.05] reflex could not be identified.

#### Blood pressure, heart rate and electrodermal activity

The test group and the control group did not significantly differ with regard to BP values (see Table 3; *p*>0.05). In both groups, blood pressure increases were only observed during BL2 of the pre-conditioning phase, where cold-water stimulation (HNCS) generated average BP increases ranging from 25 to 56 mmHg. The differences between BP values recorded during stimulation blocks without cold water immersion (baseline- and CS-related phases) and the one with ice-water immersion (BL2) were all significant (all *p*<0.05; see Table 3).

In both groups, mean HR values varied between 93 and 96.5 beats per minute (BPM) and remained stable throughout all the experimental phases. Analyses of EDA responses did not reveal any significant difference between pre- (BL1) and post-conditioning BL (BL3; Δ <200 µS in all groups).

## Discussion

To our knowledge, this is the first study to uncover that differential Pavlovian (i.e. respondent) conditioning is able to activate endogenous ‘pain inhibits pain’-like mechanisms. Associative learning processes thus seem to have the capacity to sustain HNCS-induced hypoalgesia. Our results do indeed show that after repeatedly associating a tonic noxious stimulus (i.e. cold water bath, HNCS) with a differential acoustic stimulation, the paired auditory cue (CS+) was able to attenuate the electrically induced pain sensations in the test group. This decrease in pain sensitivity was accompanied by a reduction of corrugator supercilious muscle activity. This finding is reminiscent of a previous work by Flor and co-workers [15] describing successful classical conditioning of stress-induced analgesia and inherent opioid release.

Recent imaging studies have shown that in addition to classically described spinal cord-brainstem loops, brain areas like the ACC and the amygdala are also involved in pain modulation evoked by HNCS [11], [12]. Consequently, the implication of these brain structures in both learning [44], [45], [46], [14] and pain modulation processes corroborates the hypothesized relationship between the endogenous pain control systems and associative learning of cues from an individual’s environment. Traditionally, learning processes have been claimed to be involved in the development and maintenance of pain and of pain-related behavior (for review see [16]). In contrast, the present study is devoted to the potential impact of learning on pain inhibition and hence on the potential usefulness of conditioning procedures for the treatment of pain states. Whereas Flor and co-workers [15] investigated the influence of learning on stress-induced analgesia, we focused on HNCS-activated inhibition of nociceptive processing which proved to be a handy tool to assess endogenous pain control systems in both experimental [4], [5], [6] and clinical [7], [8] settings. The strong learning effects that we identified point to a potential relevance for the development of novel psychological treatment strategies. Further support for this notion may be derived from persisting effects of stimulation procedures like acupuncture or TENS. The positive therapeutic effect of these techniques has been discussed to result from DNIC-like processes [17]. But since DNIC generally last for periods of several minutes [10], associative learning effects probably partially mediate the repeatedly proven, long-lasting therapeutic efficacy of acupuncture and TENS [19], [20].

With respect to the conditioning paradigm, it should be noted that the concurrent initiation and duration of the CS and the US might be indicative of a simultaneous conditioning procedure. It should however be taken into account that the ice water-related pain sensations typically occurred after an immersion period of about 20 seconds [36]. In fact, the onset of the CS thus preceded the HNCS-related effects by this time interval. Consequently, the learning procedure may rather be considered as delay conditioning (for review see [47]). This paradigm is commonly used as an effective tool for the conditioning of emotional reactions and requires brain regions like the ones mentioned above. It is characterized by a reduced participation of hippocampal activity which may rather encode temporal information related to time intervals passing between CS and US onset that are characteristic of trace conditioning [48].

In the present study, physiological indicators of nociceptive processing were included in addition to psychophysical parameters. In particular, we measured noxious stimulation-induced reflexes of the corrugator (frowning or brow lowering reflex) and biceps femoris muscle (RIII flexion reflex). We decided to measure corrugator activity since changes in frowning activity have been shown to be a reliable tool to assess non-verbal pain expression [2], [39], [5], with a major emphasis on the affective dimension of pain (e.g. pain unpleasantness. In this context, emotional expression (e.g. pain-related facial expression in social settings) has been shown to determine frowning reflex amplitudes [49]. These findings could account for the observed respondent conditioning effect of the frowning response. In fact, the magnitude of the facial motor behavior inhibition upon presentation of CS+ in the post-conditioning phase was comparable to the one recorded during the HNCS procedure.

Since the nocifensive RIII flexion reflex has repeatedly been assessed in studies on the DNIC phenomenon [40], [24], [50], we included it as a second objective indicator of nociceptive processing. In line with the cited previous findings, we also found a reduction of RIII-related EMG activity upon HNCS. In response to the post-conditioning CS+ presentation however, attenuations of withdrawal reflex responses were observed both in the test group and in the control group. Furthermore, the CS– induced reductions in the RIII reflex activity of the test group were quite similar to those provoked by CS+ stimulations. We can thus conclude that, contrary to our initial hypothesis, respondent conditioning had no significant influence on the RIII flexion reflex. The successful conditioning of pain perception and corrugator muscle activation and the lack of conditioning effects on RIII reflex amplitudes observed in the present study may be related to differential neural circuitry involved in the respective reactions. The nocifensive RIII reflex is known to depend mainly on segmental spinal processing to ensure rapid and reliable withdrawal from noxious stimuli [23]. Accordingly, it was found to be unchanged in paraplegic patients [51]. Corrugator muscle activity and pain sensitivity are more significantly governed by higher order brain structures like prefrontal and cingulate cortical areas and the amygdala [52], [53], which are also heavily involved in learning processes and in emotional regulation. It thus seems plausible that these structures provided a more suitable substrate for Pavlovian conditioning.

We measured BP and HR to control for potential confounding effects of baroreceptor reflex-mediated modulation of pain sensitivity triggered by repeated immersion of the hand into cold water [37], [38]. It could be observed that the blood pressure only went up during BL2, when ice-water stimulation was paired with electrical stimuli. In all the other phases and throughout the groups, it did not vary notably. The described conditioning of pain inhibition is thus not likely to be attributable to alterations in cardiovascular reactivity. We also chose to apply the constant voltage paradigm in order to provide stable electrical stimulation conditions [27], [28]. This stability was confirmed by the fact that EDA and required stimulation intensity levels remained unaltered throughout the experiment.

The conditioning effects observed in the present study could theoretically also be explained by habituation effects related to the long stimulation sequences and by a concomitant reduction in pain ratings and measures. It should however be noted that during the CS+ stimulations in the test phase, pain and corrugator muscle activity did only decrease in the test group and not in the control group that was exposed to the same number of stimuli. Moreover, no habituation time course (1/e function) of the dependent variables of interest was revealed, neither in the test group nor in the control group. As concerns potential distraction effects, the CS+ may be claimed to signal impending pain and therefore to be associated with distracting negative emotions during noxious stimulus presentation. It has been shown in this regard that negative emotions generated during unpredictable noxious (electrical) stimulations imply increases in subjective pain ratings and in absolute RIII reflex magnitudes, whereas predictable noxious stimuli induce an increase in pain sensations and a consistency in RIII reflex magnitude [54]. Our results however exhibit CS+ induced reductions in subjective ratings and decreases in RIII reflex values, which corroborate the lack of involvement of distracting emotions. It has in addition been shown that distraction does not affect RIII reflex activity induced by electrical stimulation sequences allowing for temporal summation to build up [32]. In the present study, though, the RIII reflex activity was reduced following CS+ administration while a wind-up effect was consistently realized in all stimulation blocks.

Despite our inability to provide significant data with respect to CS and group related interactions that may be due to the relatively small sample sizes, the present experimental study still provides new psychophysical and physiological evidence for the involvement of learning effects in endogenous pain control. Since our findings may be relevant for the clinical setting, further studies need to be conducted to determine the prerequisites of respondent conditioning-induced pain attenuations. Additionally, future research will have to investigate the persistence of these effects. It should finally be mentioned in the present framework that specific learning conditions could also lead to attenuated activity of endogenous pain control pathways and hence be involved in the exacerbation and chronification of pain states. Further research activities will have to be devoted to this important issue.

## References

[1] Butler RK, Finn DP (2009) Stress-induced analgesia. Prog Neurobiol 88: 184–202. 19393288 10.1016/j.pneurobio.2009.04.003

[2] Basbaum AI, Fields HL (1984) Endogenous pain control systems: brainstem spinal pathways and endorphin circuitry. Annu Rev Neurosci 7: 309–338. 6143527 10.1146/annurev.ne.07.030184.001521

[3] Yarnitsky D, Arendt-Nielsen L, Bouhassira D, Edwards RR, Fillingim RB, et al (2010) Recommendations on terminology and practice of psychophysical DNIC testing. Eur J Pain 14: 339. 20227310 10.1016/j.ejpain.2010.02.004

[4] Reinert A, Treede RD, Bromm B (2000) The pain inhibiting pain effect: an electrophysiological study in humans. Brain research 862: 103–110. 10799674 10.1016/s0006-8993(00)02077-1

[5] Streff A, Michaux G, Anton F (2011) Internal validity of inter-digital web pinching as a model for perceptual diffuse noxious inhibitory controls-induced hypoalgesia in healthy humans. Eur J Pain 15: 45–52. 20547464 10.1016/j.ejpain.2010.05.011

[6] Van Wijk G, Veldhuijzen DS (2010) Perspective on diffuse noxious inhibitory controls as a model of endogenous pain modulation in clinical pain syndromes. J Pain 11: 408–419. 20075013 10.1016/j.jpain.2009.10.009

[7] Piché M, Arsenault M, Poitras P, Rainville P, Bouin M (2010) Widespread hypersensitivity is related to altered pain inhibition processes in irritable bowel syndrome. Pain 148: 49–58. 19889500 10.1016/j.pain.2009.10.005

[8] Yarnitsky D, Crispel Y, Eisenberg E, Granovsky Y, Ben-Nun A, et al (2008) Prediction of chronic post-operative pain: pre-operative DNIC testing identifies patients at risk. Pain 138: 22–28. 18079062 10.1016/j.pain.2007.10.033

[9] Le Bars D, Dickenson AH, Besson JM (1979) Diffuse noxious inhibitory controls (DNIC). I. Effects on dorsal horn convergent neurones in the rat. Pain 6: 283–304. 460935 10.1016/0304-3959(79)90049-6

[10] Villanueva L, Le Bars D (1995) The activation of bulbo-spinal controls by peripheral nociceptive inputs: diffuse noxious inhibitory controls. Biol Res 28: 113–125. 8728826

[11] Piché M, Arsenault M, Rainville P (2009) Cerebral and cerebrospinal processes underlying counterirritation analgesia. J Neurosci 29: 14236–14246. 19906971 10.1523/JNEUROSCI.2341-09.2009 PMC6665061

[12] Sprenger C, Bingel U, Büchel C (2011) Treating pain with pain: supraspinal mechanisms of endogenous analgesia elicited by heterotopic noxious conditioning stimulation. Pain 152: 428–439. 21196078 10.1016/j.pain.2010.11.018

[13] Bush G, Luu P, Posner MI (2000) Cognitive and emotional influences in anterior cingulate cortex. Trends in cognitive sciences 4: 215–222. 10827444 10.1016/s1364-6613(00)01483-2

[14] Prévost C, McCabe JA, Jessup RK, Bossaerts P, O’Doherty JP (2011) Differentiable contributions of human amygdalar subregions in the computations underlying reward and avoidance learning. Eur J Neurosci 34: 134–145. 21535456 10.1111/j.1460-9568.2011.07686.x

[15] Flor H, Birbaumer N, Schulz R, Grüsser SM, Mucha RF (2002) Pavlovian conditioning of opioid and nonopioid pain inhibitory mechanisms in humans. Eur J Pain 6: 395–402. 12160514 10.1016/s1090-3801(02)00043-5

[16] Flor H, Turk DC (2011) The Psychology of Pain. In: Flor H, Turk DC, editors. Chronic pain: an integrated biobehavioral approach. Seattle: IASP Press. 45–88 p.

[17] Le Bars D, Willer JC, De Broucker T, Villanueva L (1988) Neurophysiological mechanisms involved in the pain-relieving effects of counter-irritation and related techniques including acupuncture. Scientific Bases of Acupuncture. Berlin: Springer Verlag. 79–112 p.

[18] Le Bars D, Willer JC (2002) Pain modulation triggered by high-intensity stimulation: Implication for acupuncture analgesia? International Congress Series 1238: 11–29.

[19] Carlsson CPO (2002) Acupuncture mechanisms for clinical long-term effects, a hypothesis. International Congress Series 1238: 31–47.

[20] Price DD, Rafii A, Watkins LR, Buckingham B (1984) A psychophysical analysis of acupuncture analgesia. Pain 19: 27–42. 6234501 10.1016/0304-3959(84)90062-9

[21] Craig KD, Patrick CJ (1985) Facial expression during induced pain. Journal of Personality and Social Psychology 48: 1080–1091. 3989673 10.1037/0022-3514.48.4.1089

[22] Micalos PS, Drinkwater EJ, Cannon J, Arendt-Nielsen L, Marino FE (2009) Reliability of the nociceptive flexor reflex (RIII) threshold and association with pain threshold. Eur J Appl Physiol 105: 55–62. 18818941 10.1007/s00421-008-0872-x

[23] Sandrini G, Serrao M, Rossi P, Romaniello A, Cruccu G, et al (2005) The lower limb flexion reflex in humans. Prog Neurobiol 77: 353–395. 16386347 10.1016/j.pneurobio.2005.11.003

[24] Serrao M, Rossi P, Sandrini G, Parisi L, Amabile GA, et al (2004) Effects of diffuse noxious inhibitory controls on temporal summation of the RIII reflex in humans. Pain 112: 353–360. 15561391 10.1016/j.pain.2004.09.018

[25] Charlton E (1995) Ethical guidelines for pain research in humans. Committee on Ethical Issues of the International Association for the Study of Pain. Pain 63: 277. 8719527 10.1016/0304-3959(95)90040-3

[26] al’Absi M, Petersen KL, Wittmers LE (2000) Blood pressure but not parental history for hypertension predicts pain perception in women. Pain 1: 61–68. 10.1016/S0304-3959(00)00306-7 11098100

[27] Bini G, Cruccu G, Manfredi M (1981) Acute experimental dental pain: a technique for evaluating pain modulating procedures. J Neurosci Methods 3: 301–309. 6783786 10.1016/0165-0270(81)90066-2

[28] Schaefer F, Boucsein W (2000) Comparison of electrodermal constant voltage and constant current recording techniques using the phase angle between alternating voltage and current. Psychophysiology 37: 85–91. 10705770

[29] Willer JC, Roby A, Le Bars D (1984) Psychophysical and electrophysiological approaches to the pain-relieving effects of heterotopic nociceptive stimuli. Brain 107: 1095–1112. 6509310 10.1093/brain/107.4.1095

[30] Plaghki L, Bragard D, Le Bars D, Willer JC, Godfraind JM (1998) Facilitation of a nociceptive flexion reflex in man by non-noxious radiant heat produced by a laser. J Neurophysiol 79: 2557–2567. 9582228 10.1152/jn.1998.79.5.2557

[31] Arendt-Nielsen L, Brennum J, Sindrup S, Bak P (1994) Electrophysiological and psychophysical quantification of temporal summation in the human nociceptive system. Eur J Appl Physiol Occup Physiol 68: 266–273. 8039524 10.1007/BF00376776

[32] Ruscheweyh R, Kreusch A, Albers C, Sommer J, Marziniak M (2011) The effect of distraction strategies on pain perception and the nociceptive flexor reflex (RIII reflex). Pain 152: 2662–2671. 21925793 10.1016/j.pain.2011.08.016

[33] Mitchell LA, MacDonald RAR, Brodie EE (2004) Temperature and the cold pressor test. The Journal of Pain 5: 233–237. 15162346 10.1016/j.jpain.2004.03.004

[34] Rainoldi A, Melchiorri G, Caruso I (2004) A method for positioning electrodes during surface EMG recordings in lower limb muscles. J Neurosci Methods 4: 37–43. 10.1016/j.jneumeth.2003.10.014 15102501

[35] Fridlund AJ, Cacioppo JT (1986) Guidelines for human electromyographic research. Psychophysiology 23: 567–589. 3809364 10.1111/j.1469-8986.1986.tb00676.x

[36] Wolf S, Hardy JD (1941) Studies on pain. Observations on pain due to local cooling and on factors involved in the “cold pressor” effect. Journal of Clinical Investigation 20: 521. 16694857 10.1172/JCI101245 PMC435082

[37] Bruehl S, Chung OY (2004) Interactions between the cardiovascular and pain regulatory systems: an updated review of mechanisms and possible alterations in chronic pain. Neurosci Biobehav Rev 28: 5–414. 10.1016/j.neubiorev.2004.06.004 15341037

[38] Streff A, Kuehl LK, Michaux G, Anton F (2010) Differential physiological effects during tonic painful hand immersion tests using hot and ice water. Eur J Pain 14: 266–272. 19540783 10.1016/j.ejpain.2009.05.011

[39] Hermann C, Ziegler S, Birbaumer N, Flor H (2000) Pavlovian aversive and appetitive odor conditioning in humans: subjective, peripheral, and electrocortical changes. Exp Brain Res 132: 203–215. 10853945 10.1007/s002210000343

[40] Bouhassira D, Danziger N, Attal N, Guirimand F, Atta N (2003) Comparison of the pain suppressive effects of clinical and experimental painful conditioning stimuli. Brain 126: 1068–1078. 12690047 10.1093/brain/awg106

[41] Cramp FL, Noble G, Lowe AS, Walsh DM, Willer JC (2000) A controlled study on the effects of transcutaneous electrical nerve stimulation and interferential therapy upon the RIII nociceptive and H-reflexes in humans. Archives of physical medicine and rehabilitation 81: 324–333. 10724078 10.1016/s0003-9993(00)90079-0

[42] France CR, Suchowiecki S (1999) A comparison of diffuse noxious inhibitory controls in men and women. Pain 81: 77–84. 10353495 10.1016/s0304-3959(98)00272-3

[43] Staud R, Robinson ME, Vierck CJ, Price DD (2003) Diffuse noxious inhibitory controls (DNIC) attenuate temporal summation of second pain in normal males but not in normal females or fibromyalgia patients. Pain 101: 167–174. 12507711 10.1016/s0304-3959(02)00325-1

[44] Büchel C, Morris J, Dolan RJ, Friston KJ (1998) Brain systems mediating aversive conditioning: an event-related fMRI study. Neuron 20: 947–957. 9620699 10.1016/s0896-6273(00)80476-6

[45] Fanselow MS, Poulos AM (2005) The neuroscience of mammalian associative learning. Annu Rev Psychol 56: 207–234. 15709934 10.1146/annurev.psych.56.091103.070213

[46] Li J, Schiller D, Schoenbaum G, Phelps EA, Daw ND (2011) Differential roles of human striatum and amygdala in associative learning. Nat Neurosci 14: 1250–1252. 21909088 10.1038/nn.2904 PMC3268261

[47] Rescorla RA (1988) Behavioral studies of Pavlovian conditioning. Annu Rev Neurosci 11: 329–52. 3284445 10.1146/annurev.ne.11.030188.001553

[48] Knight DC, Cheng DT, Smith CN, Stein EA, Helmstetter FJ (2004) Neural substrates mediating human delay and trace fear conditioning. J Neurosci 24: 218–22. 14715954 10.1523/JNEUROSCI.0433-03.2004 PMC6729570

[49] Hadjistavropoulos HD, Craig KD, Hadjistavropoulos T, Poole GD (1996) Subjective judgments of deception in pain expression: accuracy and errors. Pain 65: 251–258. 8826514 10.1016/0304-3959(95)00218-9

[50] Willer JC, Le Bars D, De Broucker T (1990) Diffuse noxious inhibitory controls in man: involvement of an opioidergic link. Eur J Pharmacol 182: 347–355. 2168836 10.1016/0014-2999(90)90293-f

[51] Sandrini G, Milanov I, Willer JC, Alfonsi E, Moglia A, et al (1999) Different effects of high doses of naloxone on spinal reflexes in normal subjects and chronic paraplegic patients. Neuroscience Letters 261: 5–8. 10081913 10.1016/s0304-3940(98)01000-3

[52] Lafate RC, Lee H, Salomons TV, Van Reekum CM, Greischer LL, et al (2012) Amygdalar function reflects common individual differences in emotion and pain regulation success. J Cogn Neurosci 24: 148–158. 21861676 10.1162/jocn_a_00125 PMC3298185

[53] Tracey I, Mantyh PW (2007) The cerebral signature for pain perception and its modulation. Neuron 55: 377–391. 17678852 10.1016/j.neuron.2007.07.012

[54] Rhudy JL, Williams AE, McCabe KM, Rambo PL, Russell JL (2006) Emotional modulation of spinal nociception and pain: The impact of predictable noxious stimulation. Pain 126: 221–233. 16890356 10.1016/j.pain.2006.06.027

